# Metabolism of Oligosaccharides and Starch in Lactobacilli: A Review

**DOI:** 10.3389/fmicb.2012.00340

**Published:** 2012-09-26

**Authors:** Michael G. Gänzle, Rainer Follador

**Affiliations:** ^1^Department of Agricultural, Food and Nutritional Science, University of AlbertaEdmonton, AB, Canada

**Keywords:** *Lactobacillus*, metabolism, isomalto-oligosaccharides, starch, fructo-oligosaccharides, galacto-oligosaccharides, raffinose-family oligosaccharides, prebiotic

## Abstract

Oligosaccharides, compounds that are composed of 2–10 monosaccharide residues, are major carbohydrate sources in habitats populated by lactobacilli. Moreover, oligosaccharide metabolism is essential for ecological fitness of lactobacilli. Disaccharide metabolism by lactobacilli is well understood; however, few data on the metabolism of higher oligosaccharides are available. Research on the ecology of intestinal microbiota as well as the commercial application of prebiotics has shifted the interest from (digestible) disaccharides to (indigestible) higher oligosaccharides. This review provides an overview on oligosaccharide metabolism in lactobacilli. Emphasis is placed on maltodextrins, isomalto-oligosaccharides, fructo-oligosaccharides, galacto-oligosaccharides, and raffinose-family oligosaccharides. Starch is also considered. Metabolism is discussed on the basis of metabolic studies related to oligosaccharide metabolism, information on the cellular location and substrate specificity of carbohydrate transport systems, glycosyl hydrolases and phosphorylases, and the presence of metabolic genes in genomes of 38 strains of lactobacilli. Metabolic pathways for disaccharide metabolism often also enable the metabolism of tri- and tetrasaccharides. However, with the exception of amylase and levansucrase, metabolic enzymes for oligosaccharide conversion are intracellular and oligosaccharide metabolism is limited by transport. This general restriction to intracellular glycosyl hydrolases differentiates lactobacilli from other bacteria that adapted to intestinal habitats, particularly *Bifidobacterium* spp.

## Introduction

Lactobacilli have complex nutritional requirements for fermentable carbohydrates, amino acids, nucleic acids and other substrates, and derive metabolic energy from homofermentative or heterofermentative carbohydrate fermentation (Hammes and Hertel, 2006). Habitats of lactobacilli are nutrient-rich and often acidic and include plants, milk and meat, and mucosal surfaces of humans and animals (Hammes and Hertel, 2006). Intestinal microbiota are characterized by a high proportion of lactobacilli particularly in animals harboring non-secretory epithelia in the upper intestinal tract, including the crop of poultry, the pars esophagus of swine, and the forestomach or rodents and members of the Equidae family (Walter, 2008). Lactobacilli are also dominant in fermentation microbiota of a majority of food fermentations, and are applied as probiotic cultures to benefit host health (Hammes and Hertel, 2006). Owing to their association with humans, food animals, and food as well as their economic importance, they have been studied for more than a century and the physiology and genetics of their monosaccharide metabolism is well understood (Orla-Jensen, 1919; Kandler, 1983; de Vos and Vaughan, 1994; Axelsson, 2004; Makarova et al., 2006; Gänzle et al., 2007).

Oligosaccharides are defined as compounds that are composed of few (2–10) monosaccharide residues (Anonymous, 1982). The use of the term “oligosaccharides” in the current scientific literature, however, differs from this definition in a number of cases. For example, the term “fructo-oligosaccharides” is generally used to include β-(1 → 2) linked fructo-oligosaccharides, excluding the digestible disaccharide sucrose; the term “galacto-oligosaccharides” generally includes the (indigestible) β-(1 → 3 or 6) linked disaccharides and α-galactosyllactose but excludes the disaccharide lactose, which is also indigestible in a majority of humans, and melibiose; the term “isomalto-oligosaccharides” generally includes the digestible disaccharide isomaltose (Roberfroid et al., 1998; MacFarlane et al., 2008; Seibel and Buchholz, 2010). This paper will use the IUPAC definition of oligosaccharides to include the corresponding disaccharides.

Oligosaccharide metabolism is essential for ecological fitness of lactobacilli in most of their food-related and intestinal habitats (de Vos and Vaughan, 1994; Bron et al., 2004; Gänzle et al., 2007; Walter, 2008; Tannock et al., 2012). Oligosaccharides are the major carbohydrate sources in cereals, milk, fruits, and the upper intestine of animals. The metabolism of mono- and disaccharides is well understood; however, few data are available on the metabolism of higher oligosaccharides which are equally abundant in many habitats. Moreover, interest in the intestinal microbial ecology as well as the widespread commercial application of prebiotic oligosaccharide preparations has shifted the research interest from (digestible) disaccharides to (indigestible) higher oligosaccharides (Barrangou et al., 2003; Kaplan and Hutkins, 2003; Walter, 2008; Seibel and Buchholz, 2010). However, the sound description of the metabolism of higher oligosaccharides is challenging. First, the *in silico* assignment of the specificity of carbohydrate transport systems or glycosyl hydrolases is unreliable (see e.g., Thompson et al., 2008; Francl et al., 2010) and often results in questionable assignments of gene function. Second, few of the relevant higher oligosaccharides are available in purified form for use as substrate. However, the determination of the growth of lactobacilli on poorly described substrates provided little relevant information on the capacity of lactobacilli to utilize oligosaccharides as carbon source. The lack of reference compounds also impedes identification and quantification of individual compounds in a mixture of oligosaccharides with chromatographic methods. Only few studies characterized oligosaccharide preparations with regards to composition, linkage type, and degree of polymerization, or monitored the metabolism of individual compounds during growth of lactobacilli (Gopal et al., 2001; Kaplan and Hutkins, 2003; Saulnier et al., 2007; Ketabi et al., 2011; Teixeira et al., 2012). Despite the importance of oligosaccharide metabolism for the performance of lactobacilli in food fermentations and in intestinal habitats, our understanding of oligosaccharide metabolism in lactobacilli remains thus limited. Particularly the delineation of metabolism of disaccharides and higher oligosaccharides is unclear, and a comprehensive description of metabolic pathways is currently not available.

This review aims to provide an overview on oligosaccharide metabolism by lactobacilli. Hydrolysis of starch, the only polysaccharide hydrolyzed by extracellular enzymes of lactobacilli, is also discussed. Oligosaccharide fermentation is discussed on the basis of metabolic studies, the cellular location, and substrate specificity of carbohydrate transport systems, glycosyl hydrolases and phosphorylases, and the distribution of the genes coding for metabolic enzymes in selected genomes of lactobacilli. Oligosaccharide metabolism is discussed in detail for four major groups of compounds (i) starch, maltodextrins, and isomalto-oligosaccharides (IMO); (ii) fructo-oligosaccharides (FOS); (iii) β-galacto-oligosaccharides (βGOS); (iv) raffinose-family oligosaccharides (RFO) as well as α-galacto-oligosaccharides (RFO and αGOS, respectively).

## Bioinformatic Analyses of Oligosaccharide and Starch Metabolism of Lactobacilli

To assess the distribution of different oligosaccharide metabolic pathways in lactobacilli, this study identified genes related to oligosaccharide metabolism in lactobacilli by bioinformatic analyses. The analysis included 38 genomes of lactobacilli that were assembled to the chromosome level (Table 1). The selection of genomes includes representatives of the six major phylogenetic groups in the genus *Lactobacillus*, the *L. salivarius* group, the *l. delbrueckii* group, the *L. buchneri* group, the *L. plantarum* group, the *L. casei* group, the *L. reuteri* group, as well as the representatives of *L. brevis* and *L. sakei* (Hammes and Hertel, 2006). The selection of organisms includes obligately homofermentative species, facultatively heterofermentative species, and obligately heterofermentative species; which were isolates from milk, meat, cereal fermentations, vegetable fermentations, and intestinal habitats. Data were obtained from NCBI GenBank (ftp://ftp.ncbi.nih.gov/genomes/Bacteria/) on 9 September 2011 (Table 1). For each species and its associated plasmids, coding sequences were extracted, translated, and a BLAST database was built using “makeblastdb” of the NCBI Standalone BLAST+ software package (version 2.2.25; Camacho et al., 2009). The query sequences were searched in each database using “blastp” of the BLAST+ software package with the standard settings and the best match was reported. Additionally, a Smith–Waterman alignment of the query sequence with the highest match was performed using a BLOSUM62 substitution matrix. The score of this alignment was divided by the score of the alignment of the query sequence to itself resulting in a score ratio. An enzyme was marked as present in a given genome or plasmid if the score ratio is above a threshold of 0.3–0.4.

**Table 1:** **Genome and plasmid sequences of *Lactobacillus* spp. used in this study**.

Abbreviation	Organism	Genome accession no.	Plasmid accession no.
Aci1	*Lactobacillus acidophilus* 30SC	CP002559	CP002561; CP002560
Aci2	*Lactobacillus acidophilus* NCFM	CP000033	
Amy1	*Lactobacillus amylovorus* GRL 1112	CP002338	CP002612; CP002613
Amy2	*Lactobacillus amylovorus* GRL1118	CP002609	CP002610
Bre1	*Lactobacillus brevis* ATCC 367	NC_008497	CP000417
Buc19	*Lactobacillus buchneri* NRRL B-30929	CP002652	CP002653; CP002654; CP002655
Cas1	*Lactobacillus casei* ATCC 334	NC_008526	CP000424
Cas2	*Lactobacillus casei* BD-II	CP002618	CP002619
Cas3	*Lactobacillus casei* BL23	NC_010999	
Cas4	*Lactobacillus casei* LC2W	CP002616	CP002617
Cas5	*Lactobacillus casei* str. Zhang	NC_014334	CP000935
Cri1	*Lactobacillus crispatus* ST1	FN692037	
Del1	*Lactobacillus delbrueckii* subsp. *bulgaricus* 2038	CP000156	
Del2	*Lactobacillus delbrueckii* subsp. *bulgaricus* ATCC 11842	CR954253	
Del3	*Lactobacillus delbrueckii* subsp. *bulgaricus* ATCC BAA-365	CP000412	
Del4	*Lactobacillus delbrueckii* subsp. *bulgaricus* ND02	CP002341	CP002342
Fer1	*Lactobacillus fermentum* CECT 5716	CP002033	
Fer2	*Lactobacillus fermentum* IFO 3956	NC_010610	
Gas1	*Lactobacillus gasseri* ATCC 33323	CP000413	
Hel1	*Lactobacillus helveticus* DPC 4571	CP000517	
Hel2	*Lactobacillus helveticus* H10	CP002429	CP002430
Joh1	*Lactobacillus johnsonii* DPC 6026	CP002464	
Joh2	*Lactobacillus johnsonii* FI9785	FN298497	FN357112; AY862141
Joh3	*Lactobacillus johnsonii* NCC 533	AE017198	
Kef1	*Lactobacillus kefiranofaciens* ZW3	CP002764	CP002765; CP002766
Pla1	*Lactobacillus plantarum* JDM1	NC_012984	
Pla2	*Lactobacillus plantarum* subsp. *plantarum* ST-III	NC_014554	CP002223
Pla3	*Lactobacillus plantarum* WCFS1	NC_004567	CR377164; CR377165; CR377166
Reu1	*Lactobacillus reuteri* 100-23	AAPZ02000001;AAPZ02000002	NC_014553; NC_001757
Reu2	*Lactobacillus reuteri* DSM 20016	NC_009513	
Reu3	*Lactobacillus reuteri* JCM 1112	NC_010609	
Reu4	*Lactobacillus reuteri* SD2112	NC_015697	CP002848; CP002845; CP002846; CP002847
Rha1	*Lactobacillus rhamnosus* GG	NC_013198	
Rha2	*Lactobacillus rhamnosus* Lc 705	FM179323	FM179324
Sak1	*Lactobacillus sakei* 23K	CR936503	
Sal1	*Lactobacillus salivarius* CECT 5713	CP002034	CP002035; CP002036; CP002037
Sal2	*Lactobacillus salivarius* UCC118	CP000233	CP000234; AF488831; AF488832
San1	*Lactobacillus sanfranciscensis* TMW 1.1304	CP002461	CP002462; CP002463

*Criteria for selection of genomes are described in the text*.

The bioinformatic analysis of genes contributing to oligosaccharide metabolism allows an assessment of the frequency of alternative pathways for oligosaccharide metabolism, identifies genes that occur together to form a functional metabolic pathway, and delineates major and convergent or divergent metabolic strategies of lactobacilli for niche adaptation by specialized oligosaccharide metabolism. However, it does not account for silent genes or orphan genes that are not expressed or not functional (Obst et al., 1992). Moreover, carbohydrate fermentation is highly variable within strains of the same species due to the loss of plasmid encoded traits (de Vos and Vaughan, 1994) and gene acquisition by lateral gene transfer (Barrangou et al., 2003). For example, gene cassettes coding for carbohydrate utilization in *L. plantarum* are highly variable and were designated as “lifestyle cassettes” that may be added or deleted according to the requirements of specific ecological niches (Siezen and van Hylckama Vlieg, 2011).

## Metabolism of α-Glucans (Maltose, Isomaltose, Maltodextrins, Isomalto-Oligosaccharides, and Starch)

Starch is the major storage polysaccharide in cereal grains, grain legumes, and many roots and tubers (van der Maarel et al., 2002; Belitz et al., 2004). Starch is composed of amylose and amylopectin. Amylose is a linear α-(1 → 4) glucose chain with a plant-specific degree of polymerization of 200–6000. Amylopectin consists of short linear α-(1 → 4) linked chains with α-(1 → 6) linked side chains (van der Maarel et al., 2002). The degree of branching is specific for the plant origin (van der Maarel et al., 2002; Belitz et al., 2004). Amylolytic degradation of amylose by α- and β-amylase and amyloglucosidase yields α-(1 → 4) linked maltodextrins, maltose, and glucose, respectively. Hydrolysis of amylopectin requires amylopullulanase or pullulanase to cleave the α-(1 → 6) linked branching points; amylopectin hydrolysis additionally yields α-d-Glu- α-(1 → 6)-d-Glu (isomaltose) and oligosaccharides with mixed α-(1 → 4) and α-(1 → 6) linkages.

Linear or branched α-glucans with α-(1 → 2), α-(1 → 3), α-(1 → 4), and α-(1 → 6) are produced by bacteria and fungi. Examples include the predominantly α-(1 → 6) linked dextran produced by *Leuconostoc mesenteroides*, and the α-(1 → 6) and α-(1 → 4) linked polysaccharides reuteran and pullulan, produced by *L. reuteri* and *Aureobasidium pullulans*, respectively (Belitz et al., 2004; van Hijum et al., 2006; Cheng et al., 2011). Isomaltose, isomaltotriose, isomaltotetraose as well as panose [α-d-Glu-α-(1 → 6)-α-d-Glu-α-(1 → 4)-d-Glu] and glucosyl-panose occur in honey, as degradation products of dextran, or as products of glucansucrases activity (Belitz et al., 2004; van Hijum et al., 2006). IMO are commercially applied as prebiotic food ingredients (Seibel and Buchholz, 2010). Commercial IMO preparations consist predominantly of di-, tri-, and tetrasaccharides and contain panose, 6′ glucosylpanose, and 6′6′ diglucosylpanose in addition to IMO (Ketabi et al., 2011).

**Figure 1:**
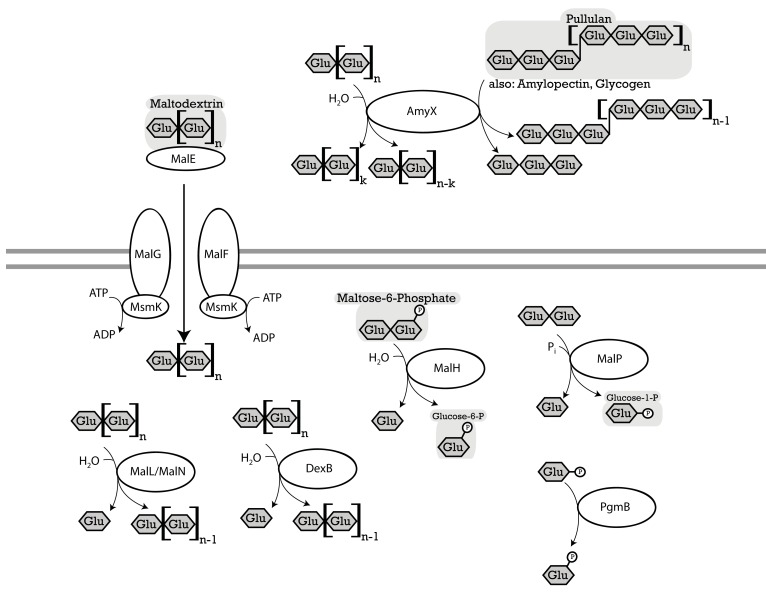
**Enzymes of lactobacilli involved in metabolism of maltose, isomaltose, maltodextrins, isomalto-oligosaccharides, and starch and their cellular location**. The distribution of genes in the genomes analyzed is shown in Table 2. **MalEFG** and**MsmK** (*L. acidophilus*), four-component ATP-binding cassette (ABC) transport system, imports maltodextrins into the cytosol (Nakai et al., 2009). **MalP** (*L. acidophilus*), named **MapA** in *L. sanfranciscensis* maltose phosphorylase, phosphorylyses maltose into d-glucose, and β-d-glucose 1-phosphate (Ehrmann and Vogel, 1998; Nakai et al., 2009). **PgmB** (*L. acidophilus*), named **PgmA** in *L. sanfranciscensis* β-phosphoglucomutase, converts β-d-glucose 1-phosphate to β-d-glucose-6-phosphate (Ehrmann and Vogel, 1998; Nakai et al., 2009). **MalH** (*L. acidophilus*), named **SimA** in *L. casei*, 6-phospho-α-glucosidase, hydrolyzes maltose-6-phosphate, trehalose-6-phosphate into d-glucose, and d-glucose-6-phosphate (Thompson et al., 1998). SimA in *L. casei* hydrolyzes the phosphorylated sucrose isomers trehalulose, turanose, maltulose, leucrose, and palatinose (Thompson et al., 2008). **AmyX** (*L. acidophilus*), **MalN** (*L. acidophilus*) and **MalL** (*L. acidophilus*), amylopullulanases, hydrolyzes α-(1 → 6)-glucosidic linkages in pullulan and amylopectin, also hydrolyzes α-(1 → 4)-glucosidic linkages in polysaccharides. AmyX a high level of similarity to *Bifidobacteria* amylopullulanase. AmyX is an extracellular enzyme, MalL and MalN are intracellular (Ryan et al., 2006; Nakai et al., 2009). MalL in *B. subtilis* also hydrolyzes sucrose (Schönert et al., 1999). **DexB** (*L. acidophilus*), α-glucosidase with activity on dextran, hydrolyzes α-(1 → 6)-glucosidic linkages, isomalto-oligosaccharides, and panose but not maltose (Møller et al., 2012).

Owing to starch hydrolysis by amylases derived from cereal grains or saliva, maltose, and maltodextrins are the most abundant oligosaccharides in cereal fermentations as well as the upper intestinal tract of grain-eating animals (Vogel et al., 1999; Tannock et al., 2012). Virtually all lactobacilli metabolize α-glucans and many strains harbor alternative pathways (Figure 1; Table 2). Moreover, amylopullulanase is the only extracellular polysaccharide hydrolyzing enzyme in lactobacilli. In lactobacilli harboring maltose phosphorylase but not hexokinase, which particularly includes strains of *L. sanfranciscensis*, maltose is the only carbohydrate that is fermented. At least two alternative systems for maltose uptake exist: (i) the ABC-transporter MalEFG/MsmK (Figure 1; Table 2); and (ii) a maltose-H^+^ symport system (Neubauer et al., 1994). A maltose phosphotransferase system has not been identified in lactobacilli. Intracellular conversion of α-glucosides occurs alternatively by the amylopullulanases MalL and MalN, the dextranase DexB, or maltose phosphorylase MalP (Figure 1; Table 2). It is noteworthy that the extracellular and intracellular amylopullulanases AmyX and MalL are homologous but differ in their cellular location (Kim et al., 2008; Møller et al., 2012).

**Table 2:** **Distribution of genes coding for metabolism of maltose, isomaltose, maltodextrins, isomalto-oligosaccharides, and starch in 38 genomes of lactobacilli**.

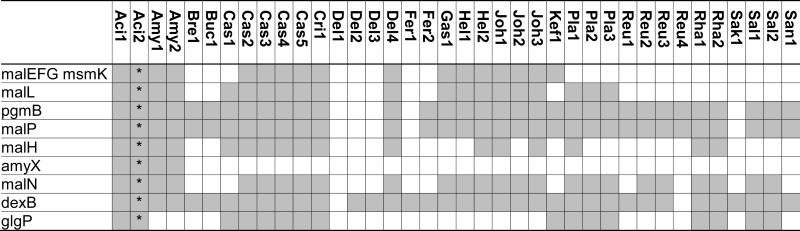

*See Figure 1 for metabolic pathways; genome accession numbers are provided in Table 1*.

Gray Background: Presence of Gene, white Background: Absence of Gene, * query sequence.

Extracellular amylase activity was characterized in several lactobacilli, including *L. fermentum*, *L. plantarum*, *L. mannihotivorans*, *L. amylovorus*, and *L. gasseri* (Giraud and Cuny, 1997; Rodriguez-Sanoja et al., 2005; Talamond et al., 2006; Kim et al., 2008). Comparable to amylolytic enzymes in bifidobacteria, extracellular amylases of lactobacilli are endoamylases hydrolyzing α-(1 → 6) as well as α-(1 → 4) glucosidic bonds in amylose, amylopectin, or pullulan. The activity increases with increasing degree of polymerization of the substrate (Talamond et al., 2006; Kim et al., 2008). Amylases activity on raw starch was dependent on the sequence of starch binding domains exhibiting significant sequence diversity (Rodriguez-Sanoja et al., 2005). Oligosaccharides with a degree of polymerization of 3 and 4 are the major products of catalysis. Amylase activity in lactobacilli is strain-specific. This study identified an extracellular amylase only in *L. acidophilus* and *L. amylovorus* (Table 2). The infrequent occurrence of amylase genes corresponds to the observation that the majority of lactobacilli are not amylolytic. However, amylolytic lactobacilli are more frequently isolated from cereal fermentations in tropical climates, possibly reflecting the lower β-amylase activity in C4 plants when compared to wheat or rye (Gänzle and Schwab, 2009; Turpin et al., 2011).

Maltose transport by the ABC-transporter MalEFG-MsmK was characterized in *L. casei* and *l. acidophilus* (Monedero et al., 2008; Nakai et al., 2009). This ABC-transporter is homologous to maltodextrin transport proteins of *Bacillus subtilis* and has a low affinity for maltose transport. Moreover, several intracellular glucanases are co-transcribed with MalEFG-MsmK, indicating the transport system functions as oligosaccharide transporter (Monedero et al., 2008; Nakai et al., 2009). The MalEFG/MsmK transport system is widespread in lactobacilli but noticeably absent in many lactobacilli that grow rapidly with maltose as the sole source of carbon (Table 2). Maltose transport in *L. sanfranciscensis* was attributed to a maltose-H^+^ symport system that was not characterized on the genetic level (Neubauer et al., 1994). Maltose phosphotransferase systems were characterized in other lactic acid bacteria (Le Breton et al., 2005) but are absent in lactobacilli (Table 2).

Three intracellular α-glucosidases hydrolyze maltodextrins or isomalto-oligosaccharides, MalL, MalN, and DexB (Figure 1; Schönert et al., 1999; Nakai et al., 2009; Møller et al., 2012). All three are GH13 enzymes. MalL in *B. subtilis* was described as sucrose-maltase-isomaltase with broad substrate spectrum (Schönert et al., 1999). Sequence homologies of MalL and MalN to AmyX as well as the amylopullulanase of bifidobacteria suggest that both enzymes also hydrolyze oligosaccharides with α-(1 → 4) and α-(1 → 6)glucosidic bonds (Kim et al., 2008; Nakai et al., 2009; Table 2). In *L. acidophilus* and *L. casei*, MalN, MalL, MalP, PgmB, and the ABC-transporter MalEFG-MsmK form a single maltodextrin operon regulated by MalR. Genes in the operon are induced by maltose and repressed by glucose (Monedero et al., 2008; Nakai et al., 2009). In most other lactobacilli harboring the ABC-transporter MalEFG-MsmK, MalN, MalL, MalP, and PgmB are also present, indicating a functional maltodextrin operon comparable to *L. acidophilus* and *L. casei* (Table 2). DexB is the most widely distributed gene coding for conversion of α-glucosides in lactobacilli (Table 2) but is not part of the maltodextrin operon (Møller et al., 2012). DexB hydrolyzes isomalto-oligosaccharides, panose, and dextran, but not maltose or sucrose (Møller et al., 2012).

Maltose phosphorylase (MalP, MapA in *L. sanfranciscensis*) catalyzes phosphorolysis of maltose to glucose and β-d-glucose-1-phosphate (Stolz et al., 1996; Ehrmann and Vogel, 1998). Maltose phosphorylase is invariably associated with β-phosphoglucomutase converting β-d-glucose-1-phosphate to glucose-6-phosphate (Table 2). Phosphorolysis of maltose does not expend ATP for generation of glucose-6-phosphate and is energetically more favorable than hydrolysis (Stolz et al., 1996). During maltose metabolism of *L. sanfranciscensis*, *L. reuteri*, and *L. fermentum*, glucose is transiently accumulated in the medium, indicating that glucose-6-phosphate is preferentially metabolized. Maltose phosphorylase is highly specific for maltose and does not convert isomaltose, kojibiose, nigerose, or maltodextrins (Ehrmann and Vogel, 1998; Nakai et al., 2009). In most obligate heterofermentative lactobacilli (Table 2), i.e., *L. brevis*, *L. buchneri*, *L. fermentum*, and *L. reuteri*, maltose phosphorylase is the only enzyme active on maltose. In other lactobacilli, maltose phosphorylase is part of the MalEFG/MsmK maltodextrin operon, together with the α-glucosidases MalL and MalN. The narrow substrate specificity of MalP implies that MalL and MalN hydrolyze maltodextrins and isomalto-oligosaccharides while MalP converts maltose, one of the products of MalN and MalL activities. Several lactobacilli harbor glycogen phosphorylase (GlgP) in addition to maltose phosphorylase (Table 2). In contrast to MalP, GlgP shows activity with maltotriose, higher maltodextrins, and glycogen but not with maltose as substrate (Alonso-Casajus et al., 2006). However, the substrate specificity of bacterial glycogen phosphorylases is poorly characterized and a contribution to carbohydrate metabolism in lactobacilli remains unclear.

The contribution of MalH, a phospho-α-glucosidase, to maltose metabolism in lactobacilli is unclear as a corresponding phosphotransferase system is lacking (Figure 1). GlvA, the corresponding phospho-α-glucosidases in *B. subtilis*, hydrolyzes maltose-6-phosphate as well as trehalose-6-phosphate (Thompson et al., 1998). The GlvA/MalH homolog SimA in *L. casei* contributes to metabolism of sucrose isomers rather than maltose (see below and Thompson et al., 2008).

Several strains of *L. reuteri* harbor GtfB, a GH70 4,-6-α-glucanotransferase active with maltodextrins as substrates. GtfB cleaves α-(1 → 4) linkages in maltotetraose to synthesize α-(1 → 6) linked oligo- and polysaccharides (Kralj et al., 2011). Glucose and maltose are among the reaction products from maltotetraose but the *in vivo* role of the enzyme may relate to polysaccharide synthesis and modification rather than oligosaccharide metabolism (Kralj et al., 2011).

## Metabolism of Sucrose and Fructo-Oligosaccharides

FOS consist of β-(2 → 1) or β-(2 → 6)-linked d-fructose units linked to a terminal d-glucose or d-fructose. Sucrose is widespread in plants and is the most abundant sugar in many fruits, grain legumes, and ungerminated cereal grains. The inulin-type β-(2 → 1) linked FOS 1-kestose, nystose, and 1-fructofuranosylnystose are also widespread in nature, although they typically occur at lower concentrations than sucrose. High concentrations are found in Jerusalem artichoke, onions, and chicory root. Wheat, rye, and barley contain 0.15–0.4% of the FOS with a degree of polymerization of 3–5 (Campbell et al., 1997). 1-Kestose is more abundant than nystose and fructofuranosylnystose in cereal grains whereas the tri-, tetra-, and pentasaccharides are approximately equally abundant in onions and chicory roots (Campbell et al., 1997). Levan-type β-(2 → 6) linked FOS, 6-kestose, and higher oligosaccharides, are less abundant in nature but are formed by degradation of levan-type fructans, or by bacterial levansucrases (Praznik et al., 1992; van Hijum et al., 2006). Inulin-type FOS are commercially applied as prebiotic food ingredients (Roberfroid et al., 1998). Commercial production of FOS relies on inulin hydrolysis or synthesis from sucrose by fructansucrases (Yun, 1996; Seibel and Buchholz, 2010).

**Figure 2:**
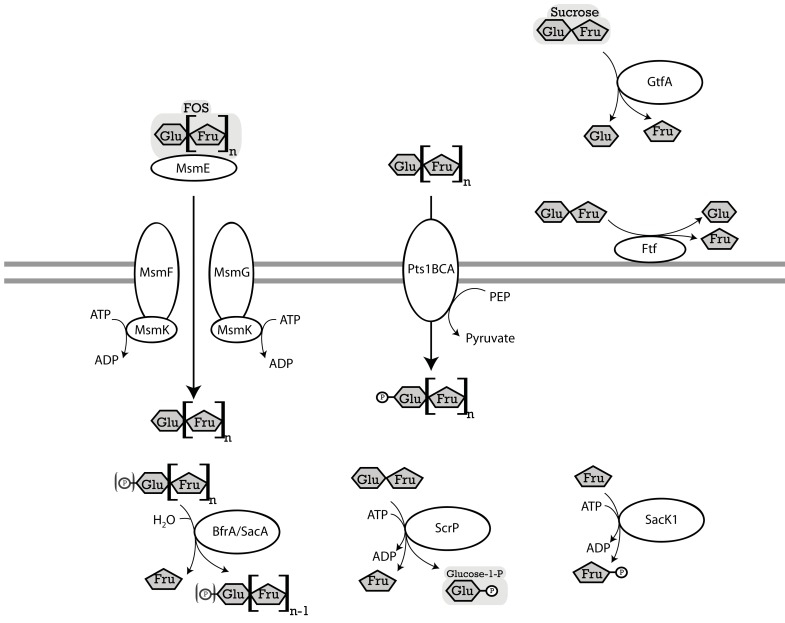
**Enzymes of lactobacilli involved in metabolism of sucrose and fructo-oligosaccharides and their cellular location**. The distribution of genes in the genomes analyzed is shown in Table 3. **MsmEFGK** (*L. acidophilus*), four-component ATP-binding cassette (ABC) transport system, imports FOS into the cytosol (Barrangou et al., 2003). ABC transport systems for FOS in lactobacilli transport glucose, fructose, and FOS with a degree of polymerization of 2–4 (Kaplan and Hutkins, 2003). **BfrA or ScrB** (*L. acidophilus*) and **SacA** (*L. plantarum*), fructosidases, hydrolyze terminal β-d-fructofuranosides in (phospho-)β-d-fructofuranoside oligosaccharides (Barrangou et al., 2003; Ehrmann et al., 2003; Saulnier et al., 2007). **ScrP** (*L. reuteri*), named **GtfA** in *L. acidophilus*, a sucrose phosphorylase, phosphorylyses sucrose to d-fructose, and α-d-glucose-1-phosphate (Barrangou et al., 2003; Schwab et al., 2007). **Pts1BCA** (*L. plantarum*), a sucrose phosphotransferase system, transports FOS into the cytosol while transferring a phosphoryl-moiety onto the glucose residue of the FOS. Pts1BCA transports FOS with a degree of polymerization of 3 and 4 (Saulnier et al., 2007). **SacK1** (*L. plantarum*), a fructokinase, transfers a phosphate group to d-fructose, converting it to a d-fructose-6-phosphate (Saulnier et al., 2007). **LevS** (*L. sanfranciscensis*) named **FtfA** in *L. reuteri*, cell-wall bound levansucrase, hydrolyzes sucrose to glucose and fructose, also has a transferase activity which catalyzes the transfer of the fructose moiety of sucrose to a fructosyl-acceptor yielding FOS or levan (Tieking et al., 2005; van Hijum et al., 2006). **GtfA** (*L. reuteri*), extracellular glucansucrase, hydrolyzes sucrose to glucose, and fructose, also has a transglucosylation activity which transfers the glucose moiety of sucrose to a glucan chain (van Hijum et al., 2006).

Three pathways for sucrose metabolism exist in lactobacilli; (i) extracellular hydrolysis by glucansucrases or fructansucrases; (ii) transport and concomitant phosphorylation of sucrose by the Pts1BCA phosphotransferase system, and hydrolysis by the (phospho-)fructo-furanosidase SacA/ScrB; and (iii) transport, followed by phosphorolysis by sucrose phosphorylase or hydrolysis by the (phospho)-fructo-furanosidases BfrA or SacA/ScrB (Figure 2; for review, see Reid and Abratt, 2005). The (phospho)-α-glucosidase MalL of *B. subtilis* also recognizes sucrose as substrate (Schönert et al., 1999) and may contribute to sucrose hydrolysis in lactobacilli. Levansucrases synthesize FOS from sucrose but do not contribute to FOS metabolism (Tieking et al., 2005; van Hijum et al., 2006). With the exception of *L. brevis*, all genomes of *Lactobacillus* spp. analyzed harbored at least one functional sucrose metabolic pathway (Table 3). The presence of two or more alternative pathways for metabolism of sucrose and higher FOS in most lactobacilli indicates that sucrose and higher FOS are highly preferred substrates. Many strains of *L. sanfranciscensis* do not metabolize sucrose; however, sucrose-negative strains *L. sanfranciscensis* in sourdough are generally associated with sucrose positive lactobacilli and thus take advantage of extracellular levansucrase activity of other strains (Tieking et al., 2003).

**Table 3:** **Distribution of genes coding for metabolism of sucrose and fructo-oligosaccharides in 38 genomes of lactobacilli**.

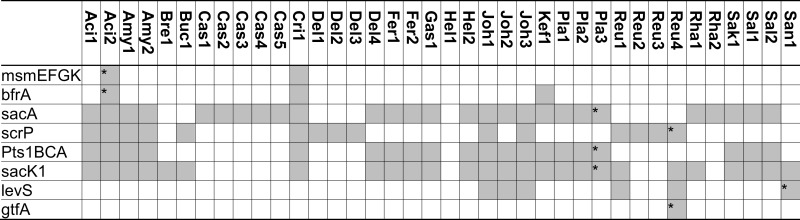

*See Figure 2 for metabolic pathways; genome accession numbers are provided in Table 1*.

Gray Background: Presence of Gene, white Background: Absence of Gene, * query sequence.

Glucansucrases and fructansucrases are the only extracellular enzymes capable of sucrose hydrolysis (Figure 2) but are the enzymes least frequently found in lactobacilli (Table 3). Glucansucrases and fructansucrases alternatively catalyze sucrose hydrolysis and oligo- or polysaccharide formation (van Hijum et al., 2006) and clearly serve ecological functions other than carbohydrate metabolism. Exopolysaccharide formation by glucansucrases and fructansucrases contributes to biofilm formation in intestinal ecosystems as well as the resistance of lactobacilli to chemical and physical stressors (Schwab and Gänzle, 2006; Walter et al., 2008). Correspondingly, their expression is induced by sucrose in some strains of *L. reuteri* but their expression in *L. reuteri* and *L. sanfranciscensis* was also reported to be constitutive or dependent on environmental stress (Tieking et al., 2005; Schwab and Gänzle, 2006; Teixeira et al., 2012). However, glucansucrases and levansucrases also contribute to sucrose metabolism. In *L. sanfranciscensis*, levansucrase is the only enzyme capable of sucrose conversion (Tieking et al., 2005; Table 3). Disruption of glucansucrases and levansucrase genes in *L. reuteri* impaired sucrose metabolism of *L. reuteri* TMW1.106 but not of *L. reuteri* LTH5448 (Schwab et al., 2007).

*In silico* and transcriptome analyses demonstrate that the functions of sucrose phosphorylase (ScrP) and (phospho-)fructo-furanonosidases (BfrA and SacA) in lactobacilli match those of sucrose phosphorylase in *L. mesenteroides*, BfrA in *Bifidobacterium lactis*, or SacA of *B. subtilis* (Kawasaki et al., 1996; Ehrmann et al., 2003; Reid and Abratt, 2005; Barrangou et al., 2006; Saulnier et al., 2007). BfrA is a fructo-furanosidase hydrolyzing sucrose, inulin-type FOS, or inulin (Ehrmann et al., 2003) that was identified in 3 of the 38 genomes analyzed (Table 3). The sucrose-(phosphate) hydrolase SacA was the most frequent fructosidase (Table 3). SacA is frequently associated with the sucrose phosphotransferase system (Table 3). The two genes are located on the same operon in *L. plantarum* and *L*. *acidophilus* and both genes are regulated by ScrR (Reid and Abratt, 2005; Barrangou et al., 2006; Saulnier et al., 2007). However, SacA of *B. subtilis* shows activity with sucrose or sucrose-1-phosphate as substrate (Reid and Abratt, 2005) and SacA is found without the associated phosphotransferase system in strains of *L. acidophilus*, *L. casei* and *L. rhamnosus* (Table 3). A SacA homolog in *Bacillus stearothermophilus* hydrolyzed sucrose but not sucrose-1-phosphate (Li and Ferenci, 1996), and SacA in *L. plantarum* hydrolyzed 1-kestose and nystose (Saulnier et al., 2007). Thus, SacA homologs in lactobacilli likely cleave FOS-phosphates as well as FOS.

Sucrose phosphorylase exhibits high specificity for sucrose as substrate (Goedl et al., 2008). Sucrose phosphorolysis is energetically more favorable than sucrose hydrolysis because glucose is phosphorylated with inorganic phosphate and not at the expense of ATP (Figure 2). However, sucrose phosphorylase was less frequently identified in genomes of lactobacilli than sucrose hydrolases (Table 3). Sucrose phosphorylase is the only intracellular sucrose converting enzyme in *L. reuteri* and significantly contributes to sucrose metabolism in this species (Schwab et al., 2007).

Transport systems for sucrose in lactobacilli include the oligosaccharide transporter MsmEFGK and the sucrose phosphotransferase system Pts1BCA (Figure 2; Barrangou et al., 2003; Saulnier et al., 2007). MsmEFGK was identified only in *L. acidophilus* and *L. crispatus* (Table 3); its presence in *L. acidophilus* was attributed to acquisition by lateral gene transfer (Barrangou et al., 2003). Both transport systems also internalize FOS with a degree of polymerization of 2 and 3 but have a very low affinity for FOS with a degree of polymerization of 4 or higher (Kaplan and Hutkins, 2003; Saulnier et al., 2007). Characterization of an ABC family transporter in *L. paracasei* demonstrated that its affinity strongly decreased in the order kestose > glucose or fructose > sucrose or nystose > fructofuranosylnystose (Kaplan and Hutkins, 2003). Irrespective of the presence of intracellular fructo-furanosidases with activity on high molecular weight fructans, transport limits metabolism of FOS in lactobacilli to di-, tri-, and tetrasaccharides. However, because many sucrose-metabolizing lactobacilli harbor neither MsmEFGK nor Pts1BCA, additional but uncharacterized sucrose transport systems exist.

## Metabolism of β-Galacto-Oligosaccharides

β-Galacto-oligosaccharides (βGOS) consist of β-(1 → 2, 3, 4, or 6) linked galactose units with terminal galactose or glucose (Torres et al., 2010; Gänzle, 2012). The only βGOS occurring widely in nature is lactose [Gal-β-(1 → 4)-Glu], which is present in the milk of mammals at concentrations of 2–10% (Gänzle et al., 2008). Tri- and tetrasaccharides (e.g., β3′ galactosyllactose, β4′ galactosyllactose, or β6′ galactosyllactose) are present only in trace amounts in humans and most non-human mammals (Kunz et al., 2000; Urashima et al., 2001; Kobata, 2010). Noticeable exceptions include the milk of some marsupials, e.g., the tammar wallaby, where β3′ galactosyllactose and corresponding higher oligosaccharides are a major component. βGOS also occur in nature as degradation products of plant galactans or arabinogalactans (Belitz et al., 2004). Moreover, βGOS are commercially produced and applied as prebiotic food ingredients (Yun, 1996; MacFarlane et al., 2008; Seibel and Buchholz, 2010). Commercial βGOS preparations have a degree of polymerization of 2–6 and contain predominantly β-(1 → 3, 4, or 6) linked di- and trisaccharides (Torres et al., 2010; Gänzle, 2012).

**Figure 3:**
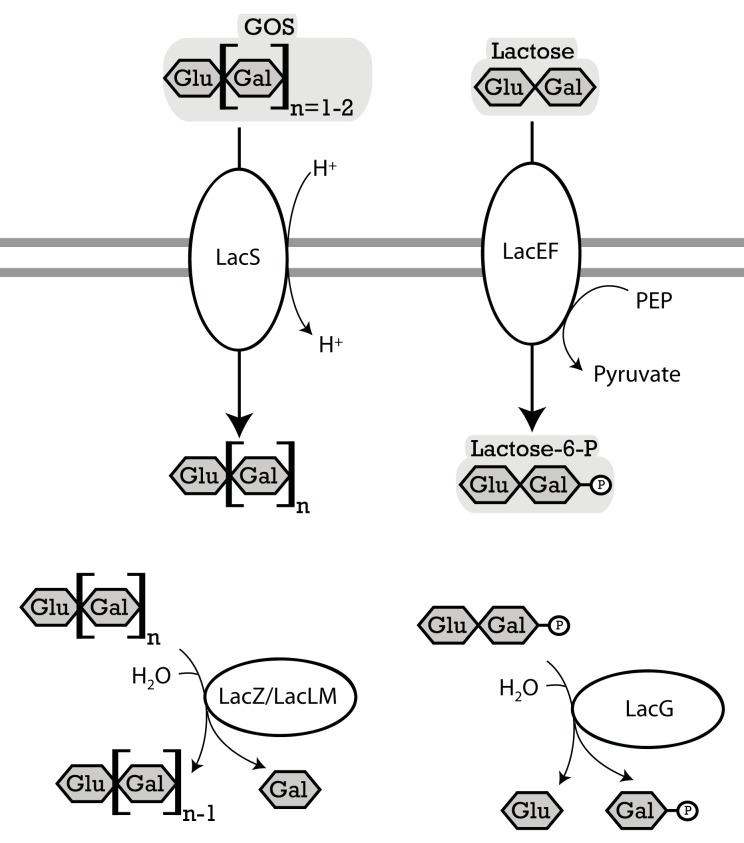
**Enzymes of lactobacilli involved in metabolism of lactose and β-galacto-oligosaccharides and their cellular location**. The distribution of genes in the genomes analyzed is shown in Table 4. **LacS** (*L. acidophilus*), named **RafP** in *L. plantarum* lactose permease, imports di- and trisaccharide GOS into the cytosol, functions either as a proton symport system or as a lactose-galactose antiporter (de Vos and Vaughan, 1994; Silvestroni et al., 2002). **LacZ** (*L. acidophilus*) and **LacLM** (*L. plantarum*), β-galactosidases, hydrolyze terminal β-d-galactose in β-d-galactosides (Schwab et al., 2010). **LacEF** (*L. casei*), a lactose phosphotransferase system, transports lactose into the cytosol while transferring a phosphoryl onto the galactose residue of the lactose (de Vos and Vaughan, 1994). **LacG** (*L. casei*), a phospho-β-galactosidase, hydrolyzes terminal units which are bound to a phospho-β-galactose (de Vos and Vaughan, 1994).

Initial studies on βGOS metabolism by lactic acid bacteria aimed to understand lactose metabolism of dairy starter cultures (Premi et al., 1972; Poolman et al., 1992; de Vos and Vaughan, 1994; Obst et al., 1995). More recent investigations on β-galactosidases of lactobacilli focused on the production and metabolism of prebiotic βGOS (e.g., Nguyen et al., 2007; Schwab et al., 2010; Andersen et al., 2011; for review, see Torres et al., 2010; Gänzle, 2012). Lactobacilli metabolize βGOS by two alternative pathways; (i) transport and concomitant phosphorylation by the LacEF phosphotransferase system and hydrolysis by LacG, a β-phospho-galactosidases, or (ii) transport by the lactose permease LacS and hydrolysis by β-galactosidase (Figure 3). The lactose permease/β-galactosidase pathway is more widespread in lactobacilli (Table 4). βGOS metabolic genes are frequently encoded on plasmids (Table 4); moreover, silent β-galactosidases were described (Obst et al., 1992). Metabolism by the LacEF/LacG exhibits several distinct characteristics from the LacS/β-galactosidase pathway. (i) The β-phospho-galactosidase LacG, classified in the GH1 family, is specific for lactose whereas organisms employing the LacS/β-galactosidase pathway also metabolize higher βGOS (Gopal et al., 2001). (ii) Organisms expressing the LacEF/LacG pathway utilize glucose and galactose simultaneously whereas metabolism by the LacS/LacLM or LacZ pathway results in preferential metabolism of glucose and excretion of galactose (de Vos and Vaughan, 1994; Francl et al., 2012). (iii) Expression of LacEF/LacG is induced by lactose, and gene expression is transcriptionally linked to galactose metabolism. In contrast, LacS and β-galactosidases in lactobacilli are often constitutively expressed, and are often located on the same genetic loci as metabolic enzymes for α-galactosides (Obst et al., 1992; de Vos and Vaughan, 1994; Silvestroni et al., 2002).

**Table 4:** **Distribution of genes coding for metabolism of lactose and β-galacto-oligosaccharides in 38 genomes of lactobacilli**.

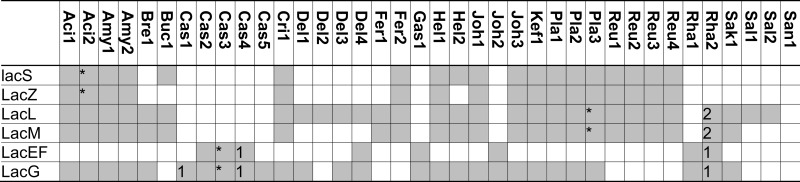

*See Figure 3 for metabolic pathways; genome accession numbers are provided in Table 1*.

Gray Background: Presence of Gene, white Background: Absence of Gene, * query sequence.

*1 Gene is present on genome and on associated plasmid, 2 Gene is present only on associated plasmid, not on genome*.

The β-galactosidases LacLM and LacZ, both classified in the GH2 family, hydrolyze a wide variety of β-(1 → 2, 3, 4, or 6) βGOS, including oligosaccharides with a degree of polymerization of 3–6 (Gänzle, 2012). LacLM or LacZ enzymes are active as multimeric enzymes (Schwab et al., 2010). The GH42 β-galactosidases LacA was cloned and characterized in *L. acidophilus* and was found to have only low activity on lactose or GOS (Schwab et al., 2010). In other GH42 family β-galactosidases from bifidobacteria and *Carnobacterium piscicola*, activity with lactose as substrate is low or absent. A contribution of LacA to βGOS metabolism in lactobacilli thus remains to be demonstrated.

βGOS are transported by the lactose permase LacS, which transports lactose in exchange with galactose, or in symport with protons (Poolman et al., 1992). Induction of *lacS* expression by βGOS with a DP of 2–6 in *L. acidophilus* was interpreted as indication that LacS transports higher βGOS as well as lactose, however, experimental evidence for tri- or tetrasaccharide transport by LacS is lacking. Lactose transport by LacS of *S. thermophilus* is inhibited by the disaccharide melibiose, indicating that αGOS are an alternative substrate for the transport enzyme (Poolman et al., 1992). *L. rhamnosus* and *L. acidophilus* were capable of acid production from galactosyllactose, but preferentially metabolized disaccharides over tri- and tetrasaccharides (Gopal et al., 2001). Because β-galactosidase exhibits no preference for disaccharides over tri- or tetrasaccharides, this preferential metabolism of βGOS with a lower degree of polymerization likely reflects transport limitations.

## Metabolism of Raffinose-Family Oligosaccharides (RFO) and α-Galacto-Oligosaccharides (α-GOS) and α-Galactosides of Lactose

The RFO raffinose, stachyose, and verbascose consist of one, two, and three α-(1 → 6) d-galactose units, respectively, linked to sucrose. RFO are widely distributed in plants, for example, ungerminated wheat and rye grains contain 0.1–0.5% raffinose; the seeds of grain legumes contain 2–10% RFO. α-Galactosides of lactose, particularly α3′ galactosyllactose, are found in the milk of several non-human mammals (Urashima et al., 2001). The αGOS melibiose [α-Gal-(1 → 6)- Glu], manninotriose [α-Gal-(1 → 6)-α-Gal-(1 → 6)-Glu], and manninotetraose occur as degradation products of RFO, or as products of transgalactosylation (Mital et al., 1973; Belitz et al., 2004; Teixeira et al., 2012).

**Figure 4:**
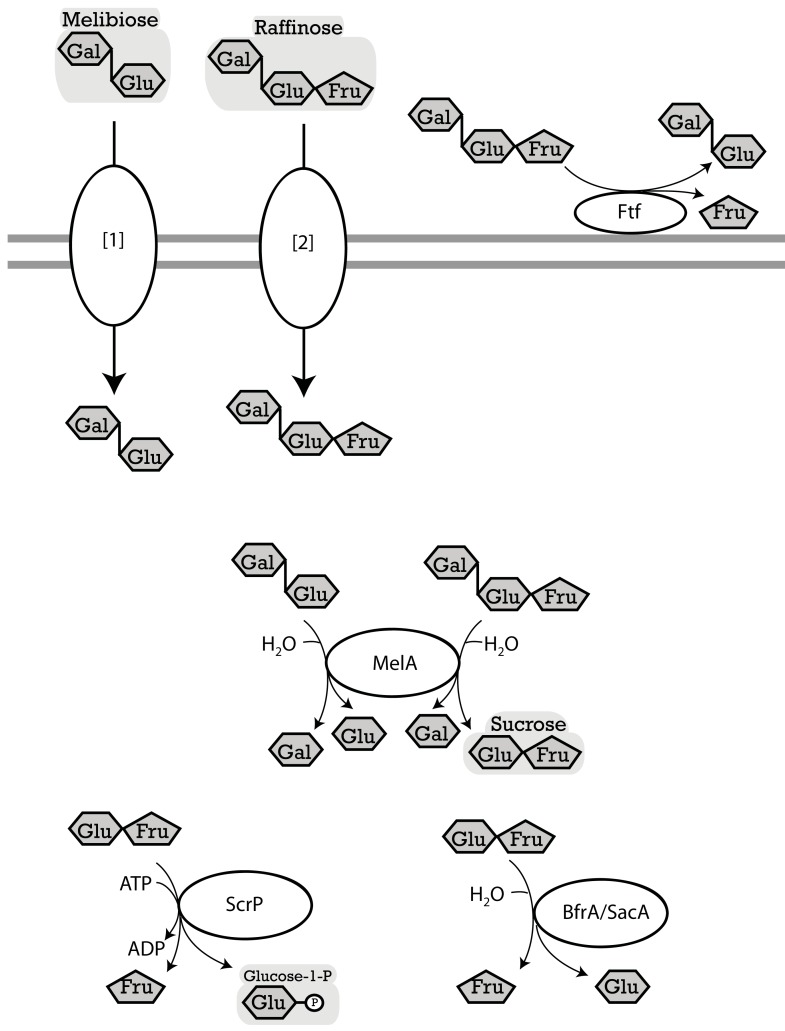
**Enzymes of lactobacilli involved in metabolism of melibiose, raffinose, and raffinose-family oligosaccharides, and their cellular location**. The distribution of genes in the genomes analyzed is shown in Table 5. **LevS** (*L. sanfranciscensis*), named **FtFA** in *L. reuteri*, cell-wall bound levansucrase, hydrolyzes sucrose, raffinose, stachyose, and verbascose to fructose and glucose, meliobiose, manninotriose, and manninotetratose, respectively. Levansucrases also have a transferase activity which catalyzes the transfer of the fructose moiety of sucrose to a fructosyl-acceptor yielding FOS or levan (Tieking et al., 2005; van Hijum et al., 2006; Teixeira et al., 2012). **MelA** (*L. acidophilus*), α-galactosidase, hydrolyzes terminal α-d-galactose residues in α-d-galactosides (Fredslund et al., 2011). **ScrP** (*L. reuteri*), named **GtfA** in *L. acidophilus*, a sucrose phosphorylase, phosphorylyses sucrose resulting from raffinose hydrolysis by MelA (Barrangou et al., 2003; Schwab et al., 2007). **[1][2]**, import of melibiose and raffinose into cytosol. Transport enzymes of lactobacilli specific for melibiose or raffinose were not characterized biochemically; candidates include **LacS** and **MelB**. LacS (lactose transport protein, ***rafP*** in *L. plantarum*) was shown in *Streptococcus thermophilus* to have an affinity not only for β-galactosides and galactose but also for melibiose and to a lesser extent for raffinose (Poolman et al., 1992; Silvestroni et al., 2002). *L. plantarum* transports melibiose independent of LacS, a possible candidate gene is the predicted melibiose permease with homology to MelB (Na+/Sugar symporter) in *Escherichia coli* (Tamura and Matsushita, 1992; Pourcher et al., 1995).

Different from FOS and βGOS, which are considered prebiotic oligosaccharides, RFO are considered anti-nutritive factors, causing dose-dependent flatulence, and gastro-intestinal discomfort (Oku and Nakamura, 2002). Accordingly, studies on RFO metabolism by lactobacilli mainly aimed at allowing their fermentative removal (Mital et al., 1973; de Giori et al., 2010). However, conversion of RFO to αGOS by lactobacilli (Teixeira et al., 2012) may prevent gastro-intestinal discomfort without eliminating prebiotic properties. The use of galactosidases for transgalactosylation, which was a focus of recent research on β-galactosidases, was also explored for α-galactosidases (Tzortzis et al., 2004). α-Galactosidase activity of lactobacilli was initially described by Mital et al. (1973). α-Galactosidase of *L. plantarum* and *L. acidophilus* is encoded by *melA*, a glycosyl hydrolase in the GH36 family (Figure 4). MelA is widely distributed in lactobacilli (Table 5), reflecting the relevance of α-galactosides in plants and intestinal ecosystems. The enzyme is active as homotetramer, and recognizes unbranched oligosaccharides, including melibiose, raffinose, and stachyose, as substrate (Silvestroni et al., 2002; Fredslund et al., 2011). Interestingly, the galactoside metabolism gene cluster in *L. plantarum* and *L. fermentum* encodes *melA* as well as a LacLM type β-galactosidase (Silvestroni et al., 2002; Carrera-Silva et al., 2006). This arrangement may reflect that melibiose rarely occurs in nature. Naturally occurring oligosaccharides with α-galactosidic bonds also contain β-galactosidic bonds (e.g., α3′ galactosyllactose) or β-(1 → 2) fructosides (e.g., raffinose). αGOS and βGOS are transported by LacS/RafP, the lactose transport protein that also shows affinity for melibiose (Poolman et al., 1992; Silvestroni et al., 2002). However, melibiose transport and utilization in mutants of *L. plantarum* that are deficient in lactose transport demonstrates that alternative transport enzymes exist (Tamura and Matsushita, 1992), potentially homologs of MelB, a melibiose permease characterized in *E. coli* (Pourcher et al., 1995). α-Galactosidase activity was initially characterized in cells grown with glucose (Mital et al., 1973); *melA* expression in *L. plantarum* is induced by melibiose but not repressed by glucose (Silvestroni et al., 2002).

**Table 5:** **Distribution of genes coding for metabolism of melibiose, raffinose, and raffinose-family oligosaccharides in 38 genomes of lactobacilli**.

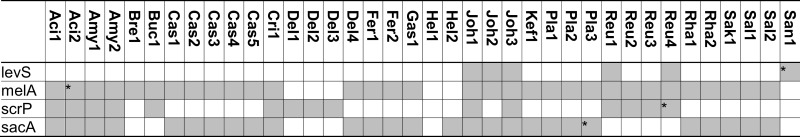

*See Figure 4 for metabolic pathways; genome accession numbers are provided in Table 1*.

Gray Background: Presence of Gene, white Background: Absence of Gene, * query sequence.

Metabolism of oligosaccharides with mixed α- and β-galactosidic linkages requires combined activity of α-galactosidase and β-galactosidase (Figures 3 and 4). Likewise, hydrolysis of RFO by α-Gal releases sucrose and complete degradation of RFO is dependent on sucrose metabolic enzymes (Figure 4). Of the sucrose metabolic enzymes shown in Figure 2, levansucrase and the fructo-furanosidase BfrA also show activity with RFO as substrates (Ehrmann et al., 2003; van Hijum et al., 2006; Teixeira et al., 2012). Few strains of lactobacilli harbor levansucrase but not α-Gal, e.g., *L. sanfranciscensis* LTH2590. These strains convert RFO to αGOS by levansucrase activity without further metabolism of the galactosides (Table 5; Teixeira et al., 2012). Glucansucrases and sucrose phosphorylase do not cleave RFO (Kim et al., 2003; van Hijum et al., 2006). In strains expressing *melA*, levansucrase, and sucrose phosphorylase or fructo-furanosidase, two alternative pathways for RFO degradation exist: (i) extracellular conversion of RFO to the corresponding αGOS and fructose by levansucrase, followed by αGOS uptake and hydrolysis; and (ii) RFO uptake, followed by hydrolysis to sucrose and galactose through MelA activity, and sucrose conversion by sucrose phosphorylase or fructo-furanosidase (Teixeira et al., 2012). Extracellular conversion by levansucrase is the preferred metabolic route in *L. reuteri* (Teixeira et al., 2012), presumably because of facilitated transport. However, raffinose induces the expression of sucrose phosphorylase in *L. reuteri* (Teixeira et al., 2012) and *B. lactis* (Trindade et al., 2003), demonstrating that both pathways exist in parallel.

## Metabolism of Trehalose, Cellobiose, and Human Milk Oligosaccharides

Lactobacilli also have the strain- or species-specific ability to metabolize the disaccharides cellobiose [Glu-β-(1 → 4)-d-Glu], gentiobiose [Glu-β-(1 → 6)-d-Glu], trehalose [Glu-α-(1 → 1)-d-α-Glu], and the α-d-glucosyl-d-fructose isomers trehalulose, turanose, maltulose, leucrose, and palatinose. Trehalose is produced in response to osmotic stress, or to survive dehydration by organisms in all kingdoms (Crowe et al., 2001). Cellobiose is the degradation product of cellulose or related plant β-glucans. Sucrose isomers, particularly leucrose, may occur in nature as a product of bacterial glucansucrase activity (van Hijum et al., 2006).

Diverse disaccharide hydrolases or disaccharide-phosphate hydrolases are found in *L. plantarum*, *L. johnsonii*, *L. casei*, and *L. acidophilus* while *L. brevis*, *L. reuteri*, and *L. delbrueckii* have a much more restricted spectrum of disaccharide-(phosphate) hydrolases (Andersson et al., 2005; Barrangou et al., 2006; Thompson et al., 2008; Francl et al., 2010). However, it was noted that the annotated specificity of sugar transport systems is generally inadequate and few studies provide functional analyses of disaccharide metabolism (Thompson et al., 2008; Francl et al., 2010). Moreover, studies on the metabolism of corresponding higher oligosaccharides in lactobacilli are lacking. Trehalose, cellobiose, and α-d-glucosyl-d-fructose isomers are all metabolized by phosphotransferase systems and intracellular phospho-glycosyl hydrolases (Barrangou et al., 2006; Thompson et al., 2008; Francl et al., 2010). Corresponding to other phosphotransferase systems, the expression of the operons is induced by the respective substrates. The spectrum of disaccharides that is metabolized by individual phosphotransferase systems can be quite diverse. For example, the phospho-α-glucosyl hydrolase in the sucrose isomer metabolism (SIM) operon of *L. casei* recognizes the five α-linked sucrose isomers (see above) as well as maltose-6-phosphate, isomaltose-6-phosphate, and trehalose-6-phosphate as substrates.

*Lactobacillus plantarum* and *L. gasseri* harbor several systems for transport and metabolism of β-glucosides (Andersson et al., 2005; Francl et al., 2010). Bioinformatics analyses predicted the presence of two β-glucoside/cellobiose specific phosphotransferase systems with associated phospho-β-glucosidase in addition to a β-glucosidase in *L. plantarum* WCFS1 (Andersson et al., 2005). It remains to be established whether this multitude of transport and enzyme systems reflects adaptation to substrates differing in their linkage type or degree of polymerization.

Human milk contains about 1% oligosaccharides. Human milk oligosaccharides consist of d-glucose, d-galactose, *N*-acetylglucosamine, *L*-fucose, and sialic acid and have a degree of polymerization of 3–32. The combination of different monomers, linkage types, and different degrees of branching or polymerization allows for a vast number of different structures. More than 100 different structures were identified and the composition of milk oligosaccharides is dependent on the mother (Kunz et al., 2000; Kobata, 2010). Human milk oligosaccharides generally consist of lactose at the reducing end and are elongated with galactose, *N*-acetylglucosamine, fucose, and sialic acid. Core structures include galactosyllactose, fucosyllactose, lacto-*N*-fucopentaose, and sialyllactose (Kunz et al., 2000; Kobata, 2010). These oligosaccharides are not degraded by β-galactosidases of lactobacilli (Schwab and Gänzle, 2011). Studies with purified human milk oligosaccharides demonstrate that lactobacilli utilize fucose and *N*-acetylglucosamine but not human milk oligosaccharides as carbon source (Ward et al., 2006; Schwab and Gänzle, 2011). Weak growth of *L. acidophilus* on human milk oligosaccharides was observed for *L. acidophilus* (Marcobal et al., 2010), which may reflect metabolism of α-galactosyllactose or β-galactosyllactose, or the ability to release few of the fucosyl- or *N*-acetylglucosaminyl-residues after cell lysis and release of intracellular glycosyl hydrolases. The inability of lactobacilli to grow on human milk oligosaccharides contrasts the metabolic toolset of bifidobacteria, which are highly adapted to growth on human milk oligosaccharides as carbon source (González et al., 2008; Sela et al., 2008).

## Conclusion

Lactobacilli are well-equipped to metabolize oligosaccharides that occur in their habitats, including plants, milk, and the (upper) intestinal tract of humans and animals. This metabolic diversity is remarkable for a group of organisms that have evolved by reduction of genome size (Makarova et al., 2006). Metabolic pathways for disaccharide metabolism often also enable the metabolism of tri- and tetrasaccharides. However, with the exception of amylase and levansucrase, metabolic enzymes for oligosaccharide conversion are intracellular and oligosaccharide metabolism is limited by transport. Starch and related α-glucans are the only group of compounds for which a metabolic pathway dedicated to oligo- and polysaccharide metabolism was retained. This general restriction to intracellular glycosyl hydrolases clearly differentiates lactobacilli from other bacteria that adapted to intestinal habitats, particularly *Bifidobacterium* spp., which maintain a more extensive toolset for extracellular hydrolysis and transport of complex carbohydrates (Sela et al., 2008; van den Broek et al., 2008). The divergent approach of bifidobacteria and lactobacilli to carbohydrate fermentation may reflect their respective dominance in the human colon, characterized by a limited availability of mono- and disaccharides, and the upper intestinal tract of animals, which offers a rich supply of oligosaccharides (Sela et al., 2008; Walter, 2008).

The capacity of individual strains and species of lactobacilli for oligosaccharide metabolism differs substantially. This metabolic diversity conforms to the phylogenetic diversity in the genus *Lactobacillus*. Several species metabolize a large diversity of different carbon sources, including all major categories of oligosaccharides. Well-characterized representatives include *L. acidophilus*, *L. casei*, and *L. plantarum*. Oligosaccharides are preferentially metabolized by phosphotransferase/phospho-glycosyl hydrolase systems and oligosaccharide metabolism is repressed by glucose (Andersson et al., 2005; Barrangou et al., 2006; Monedero et al., 2008; Francl et al., 2010). Other species in this continuum of metabolic diversity, however, exhibit more restricted carbohydrate fermentation patterns. An extreme is the “nothing but maltose or sucrose” diet of several strains of *L. sanfranciscensis*, which is partially reflected in the genome-sequenced strain *L. sanfranciscensis* TMW1.304 (Table 1). In this group of strains, oligosaccharides are preferentially metabolized by permease/phosphorylase systems and oligosaccharide metabolic enzymes are not repressed by glucose (Tieking et al., 2005; Schwab et al., 2007; Teixeira et al., 2012). Remarkably, both groups – broad versus narrow spectrum of oligosaccharide fermentation – are represented in intestinal habitats (e.g., *L. acidophilus* and *L. reuteri*) as well as food fermentations (e.g., *L. plantarum* and *L. sanfranciscensis*). Further insight into oligosaccharide metabolism in lactobacilli is dependent on the biochemical characterization of metabolic enzymes and their substrate specificity – particularly transport enzymes – and the sound quantification of oligosaccharide consumption during metabolism of lactobacilli in model substrates, and in food or intestinal ecosystems.

Glycosyl hydrolases and glycosyl phosphorylases of lactic acid bacteria have evolved as an important tool in the (chemo-)-enzymatic synthesis of functional oligosaccharides or sugar derivatives (e.g., van Hijum et al., 2006; Goedl et al., 2008; Black et al., 2012). Further insight into the diversity and catalytic properties of carbohydrate-active enzymes of lactobacilli will further improve this toolset for food-related and other applications.

## Conflict of Interest Statement

The authors declare that the research was conducted in the absence of any commercial or financial relationships that could be construed as a potential conflict of interest.

## References

[B1] Alonso-Casajus N. Dauvillé D. Miguel Viale A. Munoz F. J. Baroja-Fernández E. Moran-Zorzano M. T. Eydallin G. Ball S. Pozueta-Romero J. (2006). Glycogen phosphorylase, the product of the *glgP* gene, catalyzes glycogen breakdown by removing glucose units from the nonreducing ends in *Escherichia coli*. J. Bacteriol. 188, 5266–5272 10.1128/JB.01566-05 16816199 PMC1539952

[B2] Andersen J. M. Barrangou R. Hachem M. A. Lahtinen S. Goh Y. J. Svensson B. Klaenhammer T. R. (2011). Transcriptional and functional analysis of galactooligosaccharide uptake by *lacS* in *Lactobacillus acidophilus*. Proc. Natl. Acad. Sci. U.S.A. 108, 17785–17790 10.1073/pnas.1103060108 22006318 PMC3203779

[B3] Andersson U. Molenaar D. Radström P. de Vos W. M. (2005). Unity in organisation and regulation of catabolic operons in *Lactobacillus plantarum*, *Lactococcus lactis*, and *Listeria monocytogenes*. Syst. Appl. Microbiol. 28, 187–195 10.1016/j.syapm.2004.11.004 15900965

[B4] Anonymous. (1982). International Union of Pure and Applied Chemistry and International Union of Biochemistry, Joint commission on biochemical nomenclature: Abbreviated terminology of oligosaccharide chains. Pure Appl. Chem. 54, 1517–1522 10.1351/pac198254081517

[B5] Axelsson L. (2004). “Classification and physiology,” in Lactic Acid Bacteria: Microbiological and Functional Aspects, 3rd Edn eds Salminen S. von Wright A. Ouwehand A. (New York, NY: Marcel Dekker), 1–66

[B6] Barrangou R. Altermann E. Hutkins R. Cano R. Klaenhammer T. R. (2003). Functional and comparative genomic analyses of an operon involved in fructooligosaccharide utilization by *Lactobacillus acidophilus*. Proc. Natl. Acad. Sci. U.S.A. 100, 8957–8962 10.1073/pnas.1332765100 12847288 PMC166420

[B7] Barrangou R. Azcarate-Peril M. A. Duong T. Conners S. B. Klaenhammer T. R. (2006). Global analysis of carbohydrate utilization by *Lactobacillus acidophilus* using cDNA microarrays. Proc. Natl. Acad. Sci. U.S.A. 103, 3816–3821 10.1073/pnas.0511287103 16505367 PMC1533782

[B8] Belitz H.-D. Grosch W. Schieberle P. (2004). Food Chemistry, 3rd Edn Heidelberg: Springer

[B9] Black B. A. Lee V. S. Y. Zhao Y. Y. Hu Y. Curtis J. M. Gänzle M. G. (2012). Structural identification of novel oligosaccharides produced by *Lactobacillus bulgaricus* and *Lactobacillus plantarum*. J. Agric. Food Chem. 60, 4886–4894 10.1021/jf300917m 22497208

[B10] Bron P. A. Grangette C. Mercenier A. de Vos W. M. Kleerebezem M. (2004). Identification of *Lactobacillus plantarum* genes that are induced in the gastrointestinal tract of mice. J. Bacteriol. 186, 5721–5729 10.1128/JB.186.23.7829-7835.2004 15317777 PMC516819

[B11] Camacho C. Coulouris G. Avagyan V. Ma N. Papadopoulos J. Bealer K. Madden T. L. (2009). BLAST+: architecture and applications. BMC Bioinformatics 15, 421 10.1186/1471-2105-10-421 20003500 PMC2803857

[B12] Campbell J. M. Bauer L. L. Fahey G. C. Jr. Hogarth A. J. C. L. Wolf B. W. Hunter D. E. (1997). Selected fructooligosaccharide (1-kestose, nystose, and 1F-β-fructofuranosylnystose) composition of foods and feeds. J. Agric. Food Chem. 45, 3076–3082 10.1021/jf970087g

[B13] Carrera-Silva E. A. Silvestroni A. LeBlanc J. G. Piard J.-C. de Viori G. Sesma F. (2006). A thermostable a-galactosidase from *Lactobacillus fermentum* CRL722: genetic characterization and main properties. Curr. Microbiol. 53, 374–378 10.1007/s00284-005-0442-y 17048069

[B14] Cheng K. C. Demirci A. Catchmark J. M. (2011). Pullulan: biosynthesis, production, and applications. Appl. Microbiol. Biotechnol. 92, 29–44 10.1007/s00253-011-3477-y 21800030

[B15] Crowe J. H. Crowe L. M. Oliver A. E. Tsvetkova N. Wolkers W. Tablin F. (2001). The trehalose myth revisited: introduction to a symposium on stabilization of cells in the dry state. Cryobiology 43, 89–105 10.1006/cryo.2001.2353 11846464

[B16] de Giori G. S. Agiorre L. Marazza J. Garro M. S. (2010). “An overview of lactic acid bacteria applications for healthful soy foods development,” in Biotechnology of Lactic Acid Bacteria: Novel Applications, eds Mozzi F. Raya R. R. Vignolo G. M. (Ames, IA: Wiley-Blackwell), 289–300

[B17] de Vos W. M. Vaughan E. E. (1994). Genetics of lactose utilization in lactic acid bacteria. FEMS Microbiol. Rev. 15, 217–237 10.1111/j.1574-6976.1994.tb00136.x 7946468

[B18] Ehrmann M. A. Korakli M. Vogel R. F. (2003). Identification of the gene for β-fructofuranosidase of *Bifidobacterium lactis* DSM10140T and characterization of the enzyme expressed in *Escherichia coli*. Curr. Microbiol. 46, 391–397 10.1007/s00284-002-3908-1 12732943

[B19] Ehrmann M. A. Vogel R. F. (1998). Maltose metabolism of *Lactobacillus sanfranciscensis*: cloning and heterologous expression of the key enzymes, maltose phosphorylase and phosphoglucomutase. FEMS Microbiol. Lett. 169, 81–86 10.1111/j.1574-6968.1998.tb13302.x 9851037

[B20] Francl A. Hoeflinger J. L. Miller M. J. (2012). Identification of lactose phosphotransferase systems in *Lactobacillus gasseri* ATCC33323 required for lactose utilization. Microbiology 158, 944–952 10.1099/mic.0.052928-0 22282520

[B21] Francl A. Thongaram T. Miller M. J. (2010). The pts transporters of *Lactobacillus gasseri* ATCC 33323. BMC Microbiol. 10, 77 10.1186/1471-2180-10-77 20226062 PMC2848229

[B22] Fredslund F. Hachem M. A. Larsen J. J. Sørensen P. G. Coutinho P. M. Lo Leggio L. Svensson B. (2011). Crystal structure of α-galactosidase from *Lactobacillus acidophilus* NCFM: Insight into tetramer formation and substrate binding. J. Mol. Biol. 412, 466–480 10.1016/j.jmb.2011.07.057 21827767

[B23] Gänzle M. G. (2012). Enzymatic synthesis of galactooligosaccharides and other lactose derivatives (hetero-oligosaccharides) from lactose. Int. Dairy J. 22, 116–122 10.1016/j.idairyj.2011.06.010

[B24] Gänzle M. G. Haase G. Jelen P. (2008). Lactose – crystallisation, hydrolysis and value-added derivatives. Int. Dairy J. 18, 685–694 10.1016/j.idairyj.2008.03.003

[B25] Gänzle M. G. Schwab C. (2009). “Exploitation of the metabolic potential of lactic acid bacteria for improved quality of gluten-free bread,” in The Science of Gluten Free Foods and Beverages, eds Dal Bello F. Arendt E. (St. Paul, MN: AACC International), 99–112

[B26] Gänzle M. G. Vermeulen N. Vogel R. F. (2007). Carbohydrate, peptide and lipid metabolism of lactobacilli in sourdough. Food Microbiol. 24, 128–138 10.1016/j.fm.2006.07.006 17008155

[B27] Giraud E. Cuny G. (1997). Molecular characterization of the α-amylase genes of *Lactobacillus plantarum* A6 and *Lactobacillus amylovorus* reveals an unusual 3′end structure with direct tandem repeats and suggests a common evolutionary origin. Gene 198, 149–157 10.1016/S0378-1119(97)00309-0 9370276

[B28] Goedl C. Schwarz A. Mueller M. Brecker L. Nidetzky B. (2008). Mechanistic differences among retaining disaccharide phosphorylases: insights from kinetic analysis of active site mutants of sucrose phosphorylase and α,α-trehalose phosphorylase. Carbohydr. Res. 343, 2032–2040 10.1016/j.carres.2008.01.029 18346723

[B29] González R. Klaassens E. S. Malinen E. de Vos W. M. Vaughan E. E. (2008). Differential transcriptional response of *Bifidobacterium longum* to human milk, formula milk, and galactooligosaccharide. Appl. Environ. Microbiol. 74, 4686–4694 10.1128/AEM.01867-07 18539808 PMC2519361

[B30] Gopal P. K. Sullivan P. A. Smart J. B. (2001). Utilisation of galacto-oligosaccharides as selective substrates for growth by lactic acid bacteria including *Bifidobacterium lactis* DR10 and *Lactobacillus rhamnosus* DR20. Int. Dairy J. 11, 19–25 10.1016/S0958-6946(01)00026-7

[B31] Hammes W. P. Hertel C. (2006). The genera *Lactobacillus* and *Carnobacterium*. Prokaryotes 4, 320–403 10.1007/0-387-30744-3_10

[B32] Kandler O. (1983). Carbohydrate metabolism in lactic acid bacteria. Antonie Van Leeuwenhoek 49, 209–224 10.1007/BF00399499 6354079

[B33] Kaplan H. Hutkins R. W. (2003). Metabolism of fructooligosaccharides by *Lactobacillus paracasei* 1195. Appl. Environ. Microbiol. 69, 2217–2222 10.1128/AEM.69.4.2217-2222.2003 12676703 PMC154817

[B34] Kawasaki H. Nakamura N. Ohmori M. Sakai T. (1996). Cloning and expression in *Escherichia coli* of sucrose phosphorylase gene from *Leuconostoc mesenteroides* No. 165. Biosci. Biotechnol. Biochem. 60, 322–324 10.1271/bbb.60.322 9063982

[B35] Ketabi A. Dieleman L. Gänzle M. G. (2011). Influence of isomalto-oligosaccharides on intestinal microbiota in rats. J. Appl. Microbiol. 110, 1297–1306 10.1111/j.1365-2672.2011.04984.x 21338450

[B36] Kim J.-H. Sunako M. Ono H. Murooka Y. L. Fukusaki E. Yamashita M. (2008). Characterization of gene encoding amulopullulanase from plant-originated lactic acid bacterium, *Lactobacillus plantarum* L137. J. Biosci. Bioeng. 106, 449–459 10.1263/jbb.106.498 19111640

[B37] Kim M. Kwon T. Lee H. J. Kim K. H. Chung D. K. Ji G. E. Byeon E. S. Lee J. H. (2003). Cloning and expression of sucrose phosphorylase gene from *Bifidobacterium longum* in *E. coli* and characterization of the recombinant enzyme. Biotechnol. Lett. 25, 1211–1217 10.1023/A:1023080027754 14514069

[B38] Kobata A. (2010). Structures and application of oligosaccharides in human milk. Proc. Jpn. Acad. Ser. B Phys. Biol. Sci. 86, 731–747 10.2183/pjab.86.731 20689231 PMC3066539

[B39] Kralj S. Grijpstra P. van Leeuwen S. S. Leemhuis H. Dobruchowska J. M. van der Kaaij R. M. Malik A. Oetari A. Kamerling J. P. Dijkhuizen L. (2011). 4,6-α-Glucanotransferase, a novel enzyme that structurally and functionally provides an evolutionary link between glycoside hydrolase enzyme families 13 and 70. Appl. Environ. Microbiol. 77, 8154–8163 10.1128/AEM.05735-11 21948833 PMC3209003

[B40] Kunz C. Rudloff S. Baier W. Klein N. Stobel S. (2000). Oligosaccharides in human milk: structural, functional, and metabolic aspects. Annu. Rev. Nutr. 20, 699–722 10.1146/annurev.nutr.20.1.699 10940350

[B41] Le Breton Y. Pichereau V. Sauvageot N. Auffray Y. Rincé N. (2005). Maltose utilization in *Enterococcus faecalis*. J. Appl. Microbiol. 98, 806–813 10.1111/j.1365-2672.2004.02468.x 15752325

[B42] Li Y. Ferenci T. (1996). The *Bacillus stearothermophilus* NUB36 surA gene encodes a thermophilic sucrose related to *Bacillus subtilis* SacA. Microbiology 142, 1651–1657 10.1099/13500872-142-7-1651 8757729

[B43] MacFarlane G. T. Steed H. MacFarlane S. (2008). Bacterial metabolism and health-related effects of galacto-oligosaccharides and other prebiotics. J. Appl. Microbiol. 104, 305–344 18215222 10.1111/j.1365-2672.2007.03520.x

[B44] Makarova K. Slesarev A. Wolf Y. Sorokin A. Mirkin B. Koonin E. Pavlov A. Pavlova N. Karamychev V. Polouchine N. Shakhova V. Grigoriev I. Lou Y. Rohksar D. Lucas S. Huang K. Goodstein D. M. Hawkins T. Plengvidhya V. Welker D. Hughes J. Goh Y. Benson A. Baldwin K. Lee J. H. Díaz-Muñiz I. Dosti B. Smeianov V. Wechter W. Barabote R. Lorca G. Altermann E. Barrangou R. Ganesan B. Xie Y. Rawsthorne H. Tamir D. Parker C. Breidt F. Broadbent J. Hutkins R. O’Sullivan D. Steele J. Unlu G. Saier M. Klaenhammer T. Richardson P. Kozyavkin S. Weimer B. Mills D. (2006). Comparative genomics of lactic acid bacteria. Proc. Natl. Acad. Sci. U.S.A. 103, 15611–15616 10.1073/pnas.0607117103 17030793 PMC1622870

[B45] Marcobal A. Barboza M. Froehlich J. W. Block D. E. German J. B. Lebrilla C. B. Mills D. A. (2010). Consumption of human milk oligosaccharides by gut-related microbes. J. Agric. Food Chem. 58, 5334–5340 10.1021/jf9044205 20394371 PMC2866150

[B46] Mital B. K. Shallenberger R. S. Steinkraus K. H. (1973). α-Galactosidase activity of lactobacilli. Appl. Microbiol. 26, 783–788 4796954 10.1128/am.26.5.783-788.1973 PMC379902

[B47] Møller M. S. Fredslund F. Majumder A. Nakai H. Poulsen J.-C. N. Lo Leggio L. Svensson B. Hachem M. A. (2012). Enzymology and structure of the GH13_31 glucan 1,6-α-glucosidase that confers isomaltooligosaccharide utilization in the probiotic *Lactobacillus acidophilus*. J. Bacteriol. 194, 4249–4259 10.1128/JB.00622-12 22685275 PMC3416265

[B48] Monedero V. Yebra M. J. Poncet S. Deutscher J. (2008). Maltose transport in *Lactobacillus casei* and its regulation by inducer exclusion. Res. Microbiol. 159, 94–102 10.1016/j.resmic.2007.10.002 18096372

[B49] Nakai H. Baumann M. J. Petersen B. O. Westphal Y. Schols H. Dilokpimoi A. Hachem M. A. Lahtinen S. J. Duus J. O. Svensson B. (2009). The maltodextrin transport system and metabolism in Lactobacillus acidophilus NCFM and production of novel α-glucosides through reverse phosphorolysis by maltose phosphorylase. FEBS J. 276, 7353–7365 10.1111/j.1742-4658.2009.07445.x 19919544

[B50] Neubauer H. Glaasker E. Hammes W. P. Poolman B. Konings W. N. (1994). Mechanism of maltose uptake and glucose excretion in *Lactobacillus sanfrancisco*. J. Bacteriol. 176, 3007–3012 8188601 10.1128/jb.176.10.3007-3012.1994 PMC205458

[B51] Nguyen T.-H. Splechtna B. Yamabhai M. Haltrich D. Peterbauer C. (2007). Cloning and expression of the β-galactosidase genes from *Lactobacillus reuteri* in *Escherichia coli*. J. Biotechnol. 129, 581–591 10.1016/j.jbiotec.2007.01.034 17360065

[B52] Obst M. Hehn R. Vogel R. F. Hammes W. P. (1992). Lactose metabolism in *Lactobacillus curvatus* and *Lactobacillus sake*. FEMS Microbiol. Lett. 97, 209–214 10.1111/j.1574-6968.1992.tb05465.x

[B53] Obst M. Meding E. R. Vogel R. F. Hammes W. P. (1995). Two genes endocing the β-galactosidase of *Lactobacillus sake*. Microbiology 141, 3059–3066 10.1099/13500872-141-12-3059 8574399

[B54] Oku T. Nakamura S. (2002). Digestion, absorption, fermentation, and metabolism of functional sugar substitutes and their available energy. Pure Appl. Chem. 74, 1253–1261 10.1351/pac200274071253

[B55] Orla-Jensen S. (1919). The Lactic Acid Bacteria. Copenhagen: Høst and Søn

[B56] Poolman B. Modderman R. Reizer J. (1992). Lactose transport system of *Streptococcus thermophilus*. J. Biol. Chem. 267, 9150–9157 1577752

[B57] Pourcher T. Lelrercq S. Brandolin G. Leblanc G. (1995). Melibiose permease of *Escherichia coli*: large scale purification and evidence that H^+^, Na^+^, and Li^+^ sugar symport is catalyzed by a single polypeptide. Biochemistry 34, 4412–4420 10.1021/bi00013a033 7703254

[B58] Praznik W. Spies T. Hofinger A. (1992). Fructo-oligosaccharides from the stems of *Triticum aestivum*. Carbohydr. Res. 235, 231–238 10.1016/0008-6215(92)80091-E 1473106

[B59] Premi L. Sandine W. E. Elliker P. R. (1972). Lactose-hydrolyzing enzymes of *Lactobacillus* species. Appl. Microbiol. 24, 51–57 5057373 10.1128/am.24.1.51-57.1972 PMC380546

[B60] Reid S. J. Abratt V. R. (2005). Sucrose utilisation in bacteria: genetic organisation and regulation. Appl. Microbiol. Biotechnol. 67, 312–321 10.1007/s00253-004-1885-y 15660210

[B61] Roberfroid M. B. Van Loo I. A. Gibson G. R. (1998). The bifidogenic nature of chicory inulin and its hydrolysis products. J. Nutr. 128, 11–19 9430596 10.1093/jn/128.1.11

[B62] Rodriguez-Sanoja R. Ruiz B. Guyot J. P. Sanchez S. (2005). Starch-binding domain affect catalysis in two *Lactobacillus* α-amylases. Appl. Environ. Microbiol. 71, 297–302 10.1128/AEM.71.1.297-302.2005 15640201 PMC544272

[B63] Ryan S. M. Fitzgerald G. F. van Sinderen D. (2006). Screening for and identification of starch-, amylopectin-, and pullulan-degrading activities in bifidobacterial strains. Appl. Environ. Microbiol. 72, 5289–5296 10.1128/AEM.00257-06 16885278 PMC1538741

[B64] Saulnier D. M. A. Molenaar D. de Vos W. M. Gibson G. R. Kolida S. (2007). Identification of prebiotic fructooligosaccharide metabolism in *Lactobacillus plantarum* WCFS1 through microarrays. Appl. Environ. Microbiol. 73, 1753–1765 10.1128/AEM.01151-06 17261521 PMC1828832

[B65] Schönert S. Bruder T. Dahl M. K. (1999). Properties of maltose-inducible a-glucosidase MalL (sucrase-isomaltase-maltase) in *Bacillus subtilis*: evidence for its contribution to maltodextrin utilization. Res. Microbiol. 150, 167–177 10.1016/S0923-2508(99)80033-3 10229946

[B66] Schwab C. Gänzle M. G. (2006). Effect of membrane lateral pressure on the expression of fructosyltransferases in *Lactobacillus reuteri*. Syst. Appl. Microbiol. 29, 89–99 10.1016/j.syapm.2005.09.005 16464690

[B67] Schwab C. Gänzle M. G. (2011). Lactic acid bacteria fermentation of human milk oligosaccharide components, human milk oligosaccharides and galactooligosaccharides. FEMS Microbiol. Lett. 315, 141–148 10.1111/j.1574-6968.2010.02185.x 21175746

[B68] Schwab C. Sørensen K. I. Gänzle M. G. (2010). Heterologous expression of glycoside-hydrolase family 2 and 42 β-galactosidases of lactic acid bacteria in *Lactococcus lactis*. Syst. Appl. Microbiol. 33, 300–307 10.1016/j.syapm.2010.07.002 20822875

[B69] Schwab C. Walter J. Tannock G. W. Vogel R. F. Gänzle M. G. (2007). Sucrose utilization and impact of sucrose on glycosyltransferase expression in *Lactobacillus reuteri*. Syst. Appl. Microbiol. 30, 433–443 10.1016/j.syapm.2007.03.007 17490840

[B70] Seibel J. Buchholz K. (2010). Tools in oligosaccahride synthesis: current research and application. Adv. Carbohydr. Chem. Biochem. 63, 101–163 10.1016/S0065-2318(10)63004-1 20381705

[B71] Sela D. A. Chapman J. Adeuya A. Kim J. H. Chen F. Whitehead T. R. Lapidus A. Rokhsar D. S. Lebrilla C. B. German J. B. Price N. P. Richardson P. M. Mills D. A. (2008). The genome sequence of *Bifidobacterium longum* subsp. infantis reveals adaptations for milk utilization within the infant microbiome. Proc. Natl. Acad. Sci. U.S.A. 105, 18964–18969 10.1073/pnas.0809584105 19033196 PMC2596198

[B72] Siezen R. J. van Hylckama Vlieg J. E. T. (2011). Genomic diversity and versatility of *Lactobacillus plantarum*, a natural metabolic engineer. Microb. Cell Fact. 10 (Suppl. 1), S3 10.1186/1475-2859-10-3 21995294 PMC3271238

[B73] Silvestroni A. Connes C. Sesma F. Savoy de Giori G. Piard J.-C. (2002). Characterization of the melA locus for α-galactosidase in *Lactobacillus plantarum*. Appl. Environ. Microbiol. 68, 5464–5471 10.1128/AEM.68.11.5464-5471.2002 12406739 PMC129937

[B74] Stolz P. Hammes W. P. Vogel R. F. (1996). Maltose-phosphorylase and hexokinase activity in lactobacilli from traditionally prepared sourdoughs. Adv. Food Sci. 18, 1–6

[B75] Talamond P. Noirot M. de Kochko A. (2006). The mechanism of action of α-amylase from *Lactobacillus fermentum* on maltooligosaccharides. J. Chromatogr. B Analyt. Technol. Biomed. Life Sci. 834, 42–47 10.1016/j.jchromb.2006.02.005 16531129

[B76] Tamura C. Matsushita O. (1992). Melibiose transport system in *Lactobacillus plantarum*. Microbiol. Immunol. 36, 1119–1128 1337134 10.1111/j.1348-0421.1992.tb02116.x

[B77] Tannock G. W. Wilson C. M. Loach D. Cook G. M. Eason J. O’Toole P. W. Holtrop G. Lawley B. (2012). Resource partitioning in relation to cohabitation of *Lactobacillus* species in the mouse forestomach. ISME J. 6, 927–938 10.1038/ismej.2011.161 22094343 PMC3329185

[B78] Teixeira J. S. McNeill V. Gänzle M. G. (2012). Levansucrase and sucrose phoshorylase contribute to raffinose, stachyose, and verbascose metabolism by lactobacilli. Food Microbiol. 31, 278–284 10.1016/j.fm.2012.03.003 22608234

[B79] Thompson J. Jakubovics N. Abraham B. Hess S. Pikis A. (2008). The sim operon facilitates the transport and metabolism of sucrose isomers in *Lactobacillus casei* ATCC334. J. Bacteriol. 190, 3362–3373 10.1128/JB.02008-07 18310337 PMC2347381

[B80] Thompson J. Pikis A. Ruvinov S. B. Henrissat B. Yamamoto H. Sekiguchi J. (1998). The gene glvA of *Bacillus subtilis* 168 encodes a metal-requiring, NAD(H)-dependent 6-phospho-alpha-glucosidase. Assignment to family 4 of the glycosylhydrolase superfamily. J. Biol. Chem. 273, 27347–22756 10.1074/jbc.273.24.14788 9765262

[B81] Tieking M. Ehrmann M. A. Vogel R. F. Gänzle M. G. (2005). Molecular and functional characterization of a levansucrase from *Lactobacillus sanfranciscensis*. Appl. Microbiol. Biotechnol. 66, 655–663 10.1007/s00253-004-1773-5 15735966

[B82] Tieking M. Korakli M. Ehrmann M. A. Gänzle M. G. Vogel R. F. (2003). In situ production of EPS by intestinal and cereal isolates of lactic acid bacteria during sourdough fermentation. Appl. Environ. Microbiol. 69, 945–952 10.1128/AEM.69.2.945-952.2003 12571016 PMC143589

[B83] Torres D. P. M. do Pilar G. Gonçalves M. Teixeira J. A. Rodrigues L. R. (2010). Galacto-oligosaccharides: production, properties, applications, and significance as prebiotics. Compr. Rev. Food Sci. Food Saf. 9, 438–454 10.1111/j.1541-4337.2010.00119.x 33467830

[B84] Trindade M. I. Abratt V. R. Reid S. J. (2003). Induction of sucrose utilization genes from *Bifidobacterium lactis* by sucrose and raffinose. Appl. Environ. Microbiol. 69, 24–32 10.1128/AEM.69.1.24-32.2003 12513973 PMC152442

[B85] Turpin W. Humblot C. Guyot J.-P. (2011). Genetic screening of functional properties of lactic acid bacteria in a fermented pearl millet slurry and in the metagenome of fermented starchy foods. Appl. Environ. Microbiol. 77, 8722–8734 10.1128/AEM.05988-11 22003019 PMC3233104

[B86] Tzortzis G. Goulas A. K. Baillon M.-L. A. Gibson G. R. Rastall R. A. (2004). In vitro evaluation of the fermentation properties of galactooligosaccharides synthesized by α-galactosidase from *Lactobacillus reuteri*. Appl. Microbiol. Biotechnol. 64, 106–111 10.1007/s00253-003-1427-z 13680200

[B87] Urashima T. Saito T. Nakamura T. Messer M. (2001). Oligosaccharides of milk and colostrum in non-human mammals. Glycoconj. J. 18, 357–371 10.1023/A:1014881913541 11925504

[B88] van den Broek L. A. M. Hinz S. W. A. Beldman G. Vincken J.-P. Voragen A. G. J. (2008). *Bifidobacterium* carbohydrases – their role in breakdown and synthesis of (potential) prebiotics. Mol. Nutr. Food Res. 52, 146–163 10.1002/mnfr.200700121 18040988

[B89] van der Maarel M. J. E. C. van der Veen B. Uitdehaag J. C. M. Leemhuis H. L. Dijkhuizen L. (2002). Properties and applications of starch-converting enzymes of the α-amylase family. J. Biotechnol. 94, 137–155 10.1016/S0168-1656(01)00407-2 11796168

[B90] van Hijum S. A. Kralj S. Ozimek L. K. Dijkhuizen L. van Geel-Schutten I. G. (2006). Structure-function relationships of glucansucrase and fructansucrase enzymes from lactic acid bacteria. Microbiol. Mol. Biol. Rev. 70, 157–176 10.1128/MMBR.70.1.157-176.2006 16524921 PMC1393251

[B91] Vogel R. F. Knorr R. Müller M. R. A. Steudel U. Gänzle M. G. Ehrmann M. A. (1999). Non-dairy lactic fermentations: the cereal world. Antonie Van Leeuwenhoek 76, 403–411 10.1023/A:1002089515177 10532397

[B92] Walter J. (2008). Ecological role of lactobacilli in the gastrointestinal tract: implications for fundamental and biomedical research. Appl. Environ. Microbiol. 74, 4985–4996 10.1128/AEM.00753-08 18539818 PMC2519286

[B93] Walter J. Schwab C. Loach D. M. Gänzle M. G. Tannock G. W. (2008). Glucosyltransferase A (GtfA) and inulosucrase (Inu) of *Lactobacillus reuteri* TMW1.106 contribute to cell aggregation, in vitro biofilm formation, and colonization of the mouse gastrointestinal tract. Microbiology 154, 72–80 10.1099/mic.0.2007/010637-0 18174127

[B94] Ward R. E. Ninonuevo M. Mills D. A. Lebrilla C. B. German J. B. (2006). In vitro fermentation of breast milk oligosaccharides by *Bifidobacterium infantis* and *Lactobacillus gasseri*. Appl. Environ. Microbiol. 72, 4497–4499 10.1128/AEM.02515-05 16751577 PMC1489581

[B95] Yun J. W. (1996). Fructooligosaccharides – occurrence, preparation, and application. Enzyme Microb. Technol. 19, 107–117 10.1016/0141-0229(95)00188-3

